# Purification of Intact Plant Protoplasts by Flotation at 1g

**DOI:** 10.1100/tsw.2002.835

**Published:** 2002-05-21

**Authors:** John Graham

**Affiliations:** School of Biomolecular Sciences, Liverpool John Moores University, UK

**Keywords:** plant protoplasts, barley leaves, OptiPrep^TM^, iodixanol, density barrier

## Abstract

From a standard plant tissue digest adjusted to a density of 1.07 g/ml, protoplasts can be harvested by flotation through a low density barrier (1.03 g/ml). The delicate nature of these bodies is suited to this flotation strategy which can be carried out at 1g.

**METHOD TYPE:** extraction, isolation, purification and separation

**SUB METHOD TYPE:** centrifugation

## INTRODUCTION

Once the cellulose walls are removed from plant cells, they become sensitive to osmotic changes in the environment, shrinking or swelling in response to the surrounding medium, with subsequent changes to their buoyant densities. In the preparation of plant protoplasts, the osmolarity of the medium used to digest the plant cell walls is therefore important; use of a digest mixture whose osmolarity is 1.8× that of the living plant tissue is a widely used guideline[1].

Similar attention must be given to devising a suitable density gradient for the purification of the intact protoplasts away from the debris of the digested walls, broken protoplasts, and the digestion medium itself. OptiPrep™ can be adjusted to an appropriate osmolarity by dissolution of a suitable salt or by addition of a sorbitol or mannitol solution of known osmolarity (see Note 1). Important osmoticum components can be added while still maintaining an appropriate physiological osmolarity. The osmolarity of the gradient solutions should be the same as that of the digesting solutions and will depend on the source material. A procedure is included which not only surface sterilizes the tissue but also weakens the cuticle thus aiding protoplast release. **This protocol has been developed for wheat or barley leaves.**

## MATERIALS AND EQUIPMENT

### Sterilization

1% Sodium hypochlorite containing 0.01% (v/v) Tween-80
Serile distilled water
70% (v/v) ethanol

### Protolast Isolation

Plasmolysing solution (PS): 5 mM MES, 1 mM $KH_2PO_4$, 0.44 M D-sorbitol, 5 mM $CaCl_2$, 2 mM $MgCl_2$, 2mM $MnCl_2$, 1 mM L-arginine, 1 mM dithiothreitol (DTT), 0.1% (w/v) polyvinylpyrrolidone-10 (PVP-10), 2 mM glutathione, 2 mM L-ascorbic acid, 0.01% (w/v) soybean trypsin inhibitor, 1,300 U /ml catalase, adjusted to pH 5.7.
Digest Solution (DS): 2%(w/v) Cellulysin™ and 0.5%(w/v) macerase in PS.
Isolation Buffer (IB): as Plamsolysing solution without the catalase and trypsin inhibitor.
OptiPrep™
Working Stock (WS): dissolve 0.6 g KCl in 100 ml of OptiPrep™ (500 mOsm).

Razor blade
Nylon mesh (pore size 50–100 μm).
Shaking water bath
Petri dishes (9 cm)
Plastic conical centrifuge tubes (50 ml)
Plastic Pasteur pipette for overlayering
Low-speed (temperature-controlled) centrifuge with swinging-bucket rotor

If a sterile preparation is required, all solutions should be filter-sterilized and the relevant equipment (razor blade, mesh, tubes etc.) sterilized.

## METHODS

1. Wash the leaves sequentially in 1% sodium hypochlorite (5 min); sterile distilled water (×3), 70% ethanol (2 min) and finally sterile distilled water again (×3).
2. Place the leaf tissue in PS (50 ml/g of tissue) for 30 min at 20°C.
3. Remove the leaves from the solution and cut in to 0.5–1 mm pieces and place in 9-cm Petri dishes (2 g of tissue per dish) containing DS (10 ml/g of tissue).
4. Digest the tissue at 20°C for 3 h, with shaking at 40 rpm for the first and last 30 min.
5. After digestion filter the contents of each culture plate through the Nylon mesh. The tissue retained by the mesh may be washed off in IB; mashed lightly to release more protoplasts, and again filtered.
6. Wash the mesh through with the buffer and make up the volume of filtrate from each plate to 30 ml in 50-ml centrifuge tubes.
7. Add 7.5 ml of WS to each 30 ml of digest to make a final density of close to 1.07 g/ml (see Note 2).
8. Dilute 2 ml of WS with 20 ml of IB (approx 1.03 g/ml). Overlay the digest mixture with 10 ml of this solution (or underlay the solution with the digest mixture).
9. Finally, layer 2–3 ml of the IB on top.
10. Centrifuge the tubes at 200*g* for 4 min in a swinging-bucket rotor at 4°C (see Note 3).

11. After centrifugation, a band of material is found at the top of the medium and in the overlying buffer. The medium from this band down to the 1.03/1.07 g/ml interface is free of material. The 1.07 g/ml layer contains particulate material and there is also a pellet.
12. Harvest the band at the top using a plastic Pasteur pipette with the tip cut off to increase the size of the orifice and thus reduce damage to the delicate protoplasts.
13. The top band contains over 95% intact protoplasts, with the remainder just showing signs of lysing and releasing chloroplasts. The number of intact protoplasts remaining in the 1.07 g/ml layer is insignificant (see Notes 4–6).

## NOTES

1. For more information on the preparation of working solutions and gradient solutions of the appropriate density and osmolarity see Refs. [2,3].
2. Plant material other than wheat and barley leaves may require small modifications to the densities of the two gradient layers. When calculating the volumes of IB and WS required to produce a particular density, the densities of both solutions must be taken into account (see Note 1).
3. Centrifugation is actually not necessary for this preparation: owing to the size of the protoplasts. They will float to the top of the density barrier in about 30 min at 1*g*.
4. The yield of protoplasts from barley leaves is approx. $4 \times 10^6$/g of tissue; with wheat, the yield is significantly lower.
5. The harvested protoplasts suspension contains a small amount of 1.03 g/ml layer (about 2% Iodixanol) which has no effect whatsoever on the protoplasts.
6. The protoplasts are in high concentration and free of any residual enzyme activity, thus eliminating the need for washing. Washing any cell (but particularly delicate plant protoplasts) by pelleting and resuspending them several times is very damaging. This protocol eliminates this requirement.

## ACKNOWLEDGEMENTS

The author and TheScientificWorld wish to thank Axis-Shield PoC, AS, Oslo, Norway for their kind permission to adapt OptiPrep™ Application Sheet C18 in the preparation of this Protocol Article
